# Synthesis and biological evaluation of oxazinonaphthalene-3-one derivatives as potential anticancer agents and tubulin inhibitors

**DOI:** 10.22038/ijbms.2020.40845.9648

**Published:** 2020-11

**Authors:** Salimeh Mirzaei, Maqsudjon Qayumov, Fahimeh Gangi, Javad Behravan, Razieh Ghodsi

**Affiliations:** 1 Biotechnology Research Center, Pharmaceutical Technology Institute, Mashhad University of Medical Sciences, Mashhad, Iran; 2 Department of Medicinal Chemistry, School of Pharmacy, Mashhad University of Medical Sciences, Mashhad, Iran

**Keywords:** Anticancer activity, Molecular docking, Oxazinonaphthalene-3-one, Resistant cancer cells, Tubulin polymerization

## Abstract

**Objective(s):**

In the present study, a new series of oxazinonaphthalene-3-one analogs was designed and synthesized as novel tubulin inhibitors.

**Materials and Methods:**

The cytotoxic activity of the synthesized compounds was evaluated against four human cancer cell lines including A2780 (human ovarian carcinoma), A2780/RCIS (cisplatin resistant human ovarian carcinoma), MCF-7 (human breast cancer cells), and MCF-7/MX (mitoxantrone resistant human breast cancer cells), those compounds which demonstrated the most antiproliferative activity in the MTT test were selected to investigate their tubulin inhibition activity and their effects on cell cycle arrest (at the G2/M phase). Moreover, molecular docking studies of the selected compounds in the catalytic site of tubulin (PDB ID: 4O2B) were carried out to describe the results of biological experiments.

**Results:**

Most of our compounds exhibited signiﬁcant to moderate cytotoxic activity against four human cancer cell lines. Among them, Compounds **4d**, **5c**, and **5g**, possessing trimethoxy phenyl, showed the most antiproliferative activity with IC50 values ranging from 4.47 to 52.8 μM.

**Conclusion:**

The flow cytometric analysis of A2780 cancer cell line treated with compounds **4d**, **5c**, and **5g** showed that these compounds induced cell cycle arrest at the G2/M phase. Compound **5g**, the most antiproliferative compound, inhibited tubulin polymerization in a dose-dependent manner. Molecular docking studies of **5g** into the colchicine-binding site of tubulin displayed a possible mode of interaction between this compound and tubulin.

## Introduction

Cancer is a public health problem in the United States and many other countries. It is nowadays the second reason of death in the United States and is predicted to exceed heart diseases as the leading cause of death in the future (1). Therefore, there is a crucial need to discover and develop novel and more effective drugs to combat cancer. Although chemotherapy is the usual method for treatment of many cancer types, it fails to cure most cancer patients with advanced disease; occurrence of drug resistance is the main cause of this failure (2, 3). 

Therefore, increasing interest has been devoted to the design and discovery of more effective anticancer agents in current medicinal chemistry.

**Figure 1:**
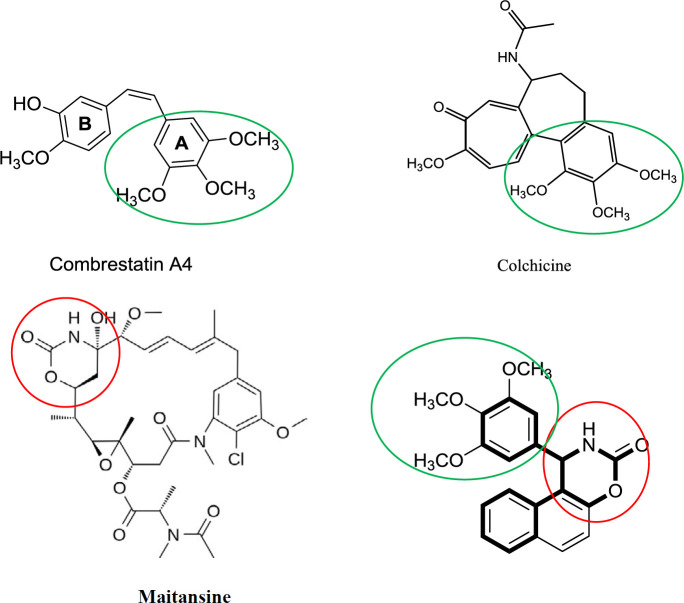
Selected tubulin inhibitors and one of our designed compounds

Microtubules play an essential role in mitosis and have long been considered an important target for the development of novel anticancer drugs. Colchicine (Figure 1) was the first drug known to bind tubulin, and it binds at a specific site called the colchicine domain (2, 3). There are a number of small molecules discovered, which bind at the colchicine site of tubulin, and hence by inhibiting microtubule polymerization cause cell cycle arrest and lead to cell death (4-8). Combretastatins, isolated from the South African tree, *Combretum caffrum*, are also a group of antimitotic compounds and combretastatin A-4 (CA-4, Figure 1) is one of the well-known natural anti-tubulin molecules, which exerts its antimitotic effect by binding to colchicine’s binding site of tubulin. The cis-double bond in CA-4 is readily converted to the more stable trans geometry during storage or use, unfortunately, this causes a dramatic decrease in both cytotoxicity and anti-tubulin activity (9-11). Many structural modifications of this compound, such as variation of substitutions on the A- and B-rings, have been reported (12-14). Numerous structure–activity relationship (SAR) studies suggested that the main pharmacophoric components are trimethoxyphenyl and p-methoxyphenyl units situated in a cis-configuration (12, 14, 15). A huge number of compounds with a modified central bridge have also been synthesized, and ultimately isomerization of the cis-double bond in CA-4 has led to extensive studies of rigid ring modifications (13-15). Maytansine (Figure 1), a benzoansamacrolide possessing 1,3-oxazinan-2-one, is a highly potent microtubule-targeted compound that induces mitotic arrest and kills tumor cells at subnanomolar concentrations(16).

In the present study, we report the design and synthesis of new compounds possessing pharmacophoric elements of antitubulins with a 1,3-oxazinan-2-one central bridge (Figure 1). The synthesized compounds were evaluated for their cytotoxic activity towards four different cancer cell lines including MCF-7, MCF-7/MX, A-2780, and A2780/RCIS. The effect of the most cytotoxic compounds to induce G2/M arrest was evaluated by flow cytometry analysis. The most cytotoxic compounds were also investigated for their activity in a microtubular polymerization assay. In addition, in order to explain the obtained biological results, docking studies have been carried out (Figure 1).

## Materials and Methods

***General chemistry***

All chemicals, reagents, and solvents used in this study were purchased from Merck AG and Aldrich Chemical. Melting points were determined with a Thomas Hoover capillary apparatus. Infrared spectra were acquired using a Perkin Elmer Model 1420 spectrometer. A Bruker FT-300 MHz instrument was used to acquire NMR spectra. Chloroform-D and DMSO-D6 were used as solvents. Coupling constant (J) values are assessed in hertz (Hz) and spin multiples are given as s (singlet), d (doublet), t (triplet), q (quartet), m (multiplet). The mass spectral measurements were performed on a 3200 QTRAP LC/MS triple quadrupole mass spectrometer with an electrospray ionization (ESI) interface.

***General procedure for preparation of 1-( substituted phenyl)-1H-naphtho[1,2-e][1,3]oxazin-3(2H)-one (4a-f)***

**Scheme 1.**
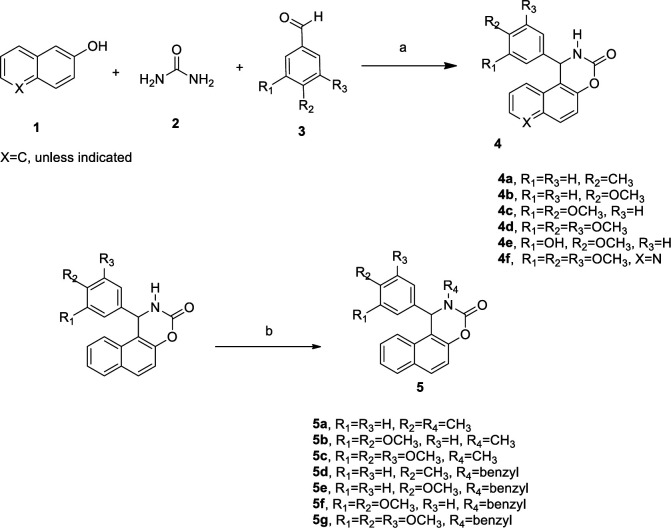
Reagents and conditions: (a) AcOH, MW (b) DMF, NaH, CH3I or benzyl bromide, rt

A mixture of β-naphthol or 6-quinolinol **1** (1 mmol), urea **2** (1.5 mmol) and substituted benzaldehydes **3** (1 mmol), and HOAc (0.3 mmol) were mixed. The reaction mixture was placed in a Pyrex test tube and irradiated for 4-20 min with a power of 700 W. After cooling, the reaction mixture was washed with water and then recrystallized from EtOAc/ Hexane (25/75) to afford the pure products **4a-4f** (Scheme 1).

***1-(p-tolyl)-1H-naphtho[1,2-e][1,3]oxazin-3(2H)-one (4a)***

Yield: 30.9%; yellow crystalline powder; mp: 173-175°C; IR(KBr): v (cm-1) 3525 (NH) 1714 (CO);1H NMR (400 MHz-DMSO): δ (ppm) 2.31 (s, 3H, CH3), 6.06-6.07 (d, 1H, CH, J=1.8 Hz), 6.16 (s, 1H, NH); 7.12-7.13 (d, 2H, ArH, J=3.6 Hz), 7.15-7.18 (d, 2H, ArH, J=9 Hz), 7.34-7.36 (d, 1H, ArH, J=6.4Hz), 7.42-7.44 (m, 2H, ArH), 7.58-7.60 (m, 1H, ArH), 7.85-7.89 (m, 2H, ArH). 

***1-(4-methoxyphenyl)-1H-naphtho[1,2-e][1,3]oxazin-3(2H)-one (4b)***

Yield: 36.21%; yellow crystalline powder; mp: 193-195°C; IR(KBr): v (cm-1) 3407 (NH), 1726 (CO);1H NMR (400 MHz-DMSO): δ (ppm) 3.77 (s, 3H, OCH3), 6.064 (d, 1H, CH, J=1.8 Hz), 6.17 (s, 1H, NH), 6.84-6.86 (d, 2H, ArH, J=6 Hz), 7.21-7.23(d, 1H, ArH, J=6 Hz), 7.32-7.34 (d, 1H, ArH, J=8.4 Hz), 7.43-7.45 (m, 3H, ArH), 7.57-7.60(m, 1H, ArH), 7.86-7.89 (m, 2H, ArH). 

***1-(3,4-dimethoxyphenyl)-1H-naphtho[1,2-e][1,3]oxazin-3(2H)-one (4c)***

Yield: 40.38%; yellow crystalline powder; mp: 178-180 °C; IR(KBr): v (cm-1) 3549 (NH), 1740 (CO);1H NMR (400 MHz-DMSO): δ (ppm) 3.67 (s, 3H, OCH3), 3.72 (s, 3H, OCH3), 6.130-6.137 (d, 1H, CH, J=2.1 Hz), 6.61-6.62 (d, 1H, ArH, J=1.5 Hz), 6.83-6.85 (d, 1H, ArH, J=6 Hz), 7.06-7.07 (d, 1H, ArH, J=3 Hz), 7.36-7.39 (d, 1H, ArH, J= 9 Hz), 7.42-7.49 (m, 2H,ArH), 7.80-7.82 (d, 1H, ArH, J=6 Hz), 7.98-8.00 (m, 2H,ArH), 8.79 (d, 1H, NH, J=1.5 Hz). 

***1-(3,4,5-trimethoxyphenyl)-1H-naphtho[1,2-e][1,3]oxazin-3(2H)-one (4d)***

Yield: 49.34%; yellow crystalline powder; mp: 216-219°C; IR(KBr): v (cm-1) 3490 (NH), 1725 (CO); 1H NMR (500 MHz-DMSO): δ (ppm) 3.55 (s, 3H, OCH3), 3.61 (s, 6H, OCH3), 6.091-6.097 (d, 1H, CH, J=1.8 Hz ), 6.56 (s, 2H, ArH), 7.33-7.34 (d, 1H, ArH, J=3 Hz), 7.40-7.43 (t, 1H, ArH, J=6 Hz), 7.46-7.49 (t, 1H, ArH, J=6 Hz), 7.82-7.84 (d, 1H, ArH, J= 6 Hz), 7.90-7.92 (d, 1H,ArH, J= 6 Hz), 7.94-7.96 (d, 1H, ArH, J= 6 Hz), 8.73-8.74 (d, 1H, NH, J=3 Hz). 

***1-(3-hydroxy-4-methoxyphenyl)-1H-naphtho[1,2-e][1,3]oxazin-3(2H)-one (4e)***

Yield: 22.97%; yellow crystalline powder; mp: 197-199°C; IR(KBr): v (cm-1)3423(NH), 1743 (CO); 1H NMR (400 MHz-DMSO): δ (ppm) 3.70 (s, 3H, OCH3), 6.042 (s, 1H, CH), 6.66-6.67(d, 1H, ArH, J=3 Hz), 6.75-6.78 (dd, 1H, ArH, J=9 Hz, J=3 Hz), 6.85-6.87 (d, 1H, ArH, J=6 Hz), 7.36-7.39 (d, 1H, ArH, J=9 Hz), 7.44-7.51 (m, 2H,ArH), 7.79-7.81 (d, 1H, ArH, J=6 Hz), 7.94-8.00 (m, 2H,ArH), 8.75 (d, 1H, NH, J=2 Hz), 9.03 (s, 1H, OH). 

***1-(3,4,5-trimethoxyphenyl)-1H-[1,3]oxazino[5,6-f]quinolin-3(2H)-one (4f)***

Yield: 36.84%; yellow crystalline powder; mp: 266-269°C;IR(KBr): v (cm-1) 3247 (NH), 1739 (CO); 1H NMR (500 MHz-DMSO): δ (ppm) 3.55 (s, 3H, OCH3), 3.62 (s, 6H, OCH3), 6.15-6.16 (d, 1H, CH, J=3 Hz ), 6.56 (s, 2H, ArH), 7.46-7.49 (m, 1H, ArH), 7.57-7.59 (d, 1H, ArH, J=6 Hz), 8.01-8.03 (d, 1H, ArH, J=61Hz), 8.28-8.30 (d, 1H, ArH, J= 6 Hz), 8.79-8.82 (m, 2H, ArH & NH); LC-MS (ESI): 366.1 (M+1)+ 

***Procedure for synthesis of 2-methyl-1-phenyl-1,2-dihydro-3H-naphtho[1,2-e][1,3]oxazin-3-one and 2-benzyl-1-phenyl-1,2-dihydro-3H-naphtho[1,2-e][1,3]oxazin-3-one (5a-g)***

To a solution of **4a-4d** (1mmol) in DMF (at 0 °C) was added sodium hydride (60% oil suspension, 3mmol), then methyl iodide or benzyl bromide (3 mmol) was added. The mixture was stirred at 0 °C for 1 hr, then reaction was continued at room temperature to afford **5a-5g **in moderate yield. The reaction mixture was diluted with water and the crude product was filtrated and recrystallized in ethanol.

***2-methyl-1-(p-tolyl)-1,2-dihydro-3H-[1,2-e][1,3]oxazin-3-one (5a)***

Yield: 59.13%; yellow crystalline powder; mp: 119-122 °C; 1H NMR (400 MHz-DMSO): δ (ppm) 2.22 (s, 3H, CH3), 2.96 (s, 3H, NCH3), 6.22 (s, 1H, CH), 7.32-7.35 (d, 2H, ArH, J=9Hz), 7.39-7.42 (d, 2H, ArH, J= 9Hz), 7.45-7.48 (dd, 1H, ArH, J=9Hz, J=0.9Hz), 7.50-7.56 (dd, 1H, ArH, J=9Hz, J=1.2Hz), 7.85-7.88 (d, 2H, ArH, J=9Hz), 7.93-8.00 (m, 2H,ArH), 13C NMR (75 MHz-DMSO): δ (ppm) 21.09, 34.63, 60.43, 115.36, 117.01, 123.25, 125.59, 127.78, 127.86, 128.90, 129.14, 130.11, 130.66, 130.88, 137.37, 138.35, 147.00, 150.03. 

***1-(3,4-dimethoxyphenyl)-2-methyl-1H-naphtho[1,2-e][1,3]oxazin-3(2H)-one (5b)***

Yield: 51.86%; yellow crystalline powder; mp: 103-105°C; 1H NMR (400 MHz-DMSO): δ (ppm) 2.98 (s, 3H, NCH3), 3.68 (s, 3H, OCH3), 3.73 (s, 3H, OCH3), 5.63 (s, 1H, CH), 6.67-6.73 (m, 2H, ArH), 6.88-6.91 (dd, 1H, ArH, J=8.4Hz, J=2Hz), 7.18-7.24 (m, 1H,ArH), 7.30-7.35 (m, 2H,ArH), 7.58-7.61 (d, 1H, ArH, J=9 Hz), 7.71-7.74 (d, 2H, ArH, J=9 Hz); 13C NMR (75 MHz-DMSO): δ (ppm) 34.73, 55.88, 55.93, 62.45, 110.13, 111.12, 114.10, 116.82, 120.48, 122.33, 125.08, 127.42, 128.88, 128.96, 130.39, 130.89, 131.74, 146.89, 149.42, 149.72, 150.32

***2-methyl-1-(3,4,5-trimethoxyphenyl)-1H-naphtho[1,2-e][1,3]oxazin-3(2H)-one (5c)***

Yield: 55.10%; yellow crystalline powder; mp: 137-140°C; 1H NMR (300 MHz-DMSO): δ (ppm) 3.00 (s, 3H, NCH3), 3.70 (s, 9H, OCH3), 5.62 (s, 1H, CH), 6.47 (s, 2H, ArH), 7.18-7.24 (m, 1H, ArH), 7.29-7.40 (m, 2H, ArH), 7.61-7.63 (d, 1H, ArH, J=8.46Hz), 7.72-7.75 (d, 2H, ArH, J= 8.46 Hz); 13C NMR (75 MHz-DMSO): δ (ppm) 34.90 ,56.10, 56.24, 60.79, 62.83, 104.75, 113.97, 116.81, 122.32, 125.16, 127.48, 128.93, 128.98, 130.52, 130.90, 134.85, 138.36, 146.97, 150.29, 153.75. 

***2-benzyl-1-(p-tolyl)-1H-naphtho[1,2-e][1,3]oxazin-3(2H)-one (5d)***

Yield: 77.74%; white crystalline powder; mp: 159-162°C; 1HNMR: 3.71 (s, 3H, CH3 ), 4.16-4.22 (d, 1H, J=16.4 Hz, CH2 ), 4.91-4.96 (d, 1H, J=16.4 Hz, CH2 ), 6.14 (s, 1H, CH ), 6.91-6.94 (d, 2H, J=9 Hz, Ar-H ), 7.26-7.34 (m, 5H, Ar-H), 7.43-7.51 (m, 5H, Ar-H), 7.85-7.87 (d, 1H, J=6 Hz, Ar-H ), 7.94-7.96 (d, 1H, J=6 Hz, Ar-H ), 7.99-8.02 (d, 1H, J=9 Hz, Ar-H ). 13CNMR:50.03, 55.57, 58.71, 114.97, 115.64, 117.13, 123.17, 125.7, 127.85, 128.01, 128.82, 129.06, 129.20, 129.30, 130.80, 130.95, 132.52, 136.88, 145.98, 146.84, 150.34, 159.69.

***2-benzyl-1-(4-methoxyphenyl)-1H-naphtho[1,2-e][1,3]oxazin-3(2H)-one (5e)***

Yield: 69.53%; white crystalline powder; mp: 163-166°C; 1HNMR: 3.72 (s, 3H, OCH3 ), 4.17-4.22 (d, 1H, J=15 Hz, CH2 ), 4.91-4.96 (d, 1H, J=15 Hz, CH2 ), 6.14 (s, 1H, CH ), 6.92-6.94 (d, 2H, J=6 Hz, Ar-H ), 7.26-7.35 (m, 5H, Ar-H), 7.43-7.54 (m, 5H, Ar-H), 7.85-7.87 (d, 1H, J=6 Hz, Ar-H ), 7.95-7.97 (d, 1H, J=6 Hz, Ar-H ), 8.00-8.03 (d, 1H, J=9 Hz, Ar-H ).13CNMR:50.04, 55.59, 58.71, 114.98, 115.64, 117.14, 123.17, 125.71, 127.85, 127.89, 128.01, 128.82, 129.07, 129.21, 129.31, 130.81, 130.95, 132.53, 136.89, 146.84, 150.34, 159.70.

***2-benzyl-1-(3,4-dimethoxyphenyl)-1H-naphtho[1,2-e][1,3]oxazin-3(2H)-one (5f)***

Yield: 72.18%; white crystalline powder; mp: 121-124°C, 1HNMR: 3.69 (s, 3H, OCH3), 3.73 (s, 3H, OCH3 ), 4.24-4.30 (d, 1H, J=16.4 Hz, CH2 ), 4.91-4.97 (d, 1H, J=16.4 Hz, CH2 ), 6.13 (s, 1H, CH ), 6.90-6.93 (d, 1H, J=9 Hz, Ar-H ), 6.97-6.99 (d, 1H, J=6 Hz, Ar-H ), 7.15 (s, 1H, NH), 7.26-7.34 (m, 5H, Ar-H), 7.43-7.54 (m, 3H, Ar-H), 7.91-7.96 (t, 2H, J=9 Hz, Ar-H ), 7.99-8.02 (d, 1H, J=9 Hz, Ar-H ). 13CNMR: 50.15, 55.92, 56.02, 59.14, 111.88, 111,95, 112.28, 112.75, 115.59, 117.11, 120.25, 123.31, 125.69, 127.87, 127.99, 128.92, 129.02, 129.16, 130.80, 130.95, 132.88, 136.98, 146.86, 149.27, 150.37.

***2-benzyl-1-(3,4,5-trimethoxyphenyl)-1H-naphtho[1,2-e][1,3]oxazin-3(2H)-one (5g)***

Yield: 79.32%; white crystalline powder; mp: 171-174°C, 1HNMR: 3.60 (s, 3H, OCH3 ), 3.72 (s, 6H, OCH3 ), 4.36-4.41 (d, 1H, J=15 Hz, CH2 ), 4.89-4.95 (d, 1H, J=15 Hz, CH2 ), 6.17 (s, 1H, CH ), 6.81 (s, 2H, Ar-H), 7.23-7.36 (m, 5H, Ar-H), 7.44-7.48 (m, 2H, Ar-H), 7.52-7.57 (t, 1H, J=6 Hz, Ar-H ), 7.94-7.97 (d, 1H, J=9 Hz, Ar-H ), 7.99-8.03 (m, 2H, Ar-H).13CNMR: 31.13, 50.53, 56.38, 59.77, 60.35, 105.39, 115.31, 117.13, 123.37, 125.75, 127.78, 127.88, 128.08, 128.91, 128.97, 129.18, 130.97, 136.16, 136.42, 137.06, 137.91, 146.97, 150.37, 153.66. 

***Biological evaluation***

*Antiproliferative activity assay*

The levels of *in vitro* proliferative responses of cancer cells and normal cells were estimated using MTT dye assay. Briefly, all stock cell lines were grown in T-75 flasks at 37 °C with 5% CO_2_ in air. Cells were seeded in a 96-well plate at a density of 5000 cells per well, with 200 µl RPMI1640 or DMEM medium for 24 hr at 37 °C in a 5% CO_2_ incubator. Then they were exposed to different concentrations of the newly synthesized compounds (compounds were prepared in concentration 0.5% DMSO and diluted with culture medium), standard anti-tumor agent CA-4, and RPMI control (no drug) for 48 hr. Thereafter, 20 µl of MTT solution (5 mg/ml) was added to each well and incubation continued at 37 °C for 3 hr. Then, the suspension was removed, and formazan crystals were solubilized in 200 µl of dimethyl sulfoxide (DMSO). Plates were shaken vigorously (400 rpm) for 5 min. The absorbance of the solution at 545 nm and 640 nm was measured using a multifunction microplate reader (Synergy H4, USA) (17-20). IC_50_ values, which were used to represent the cytotoxic effects of each compound, were calculated with nonlinear regression analysis using GraphPad Prism (version 6.)

*In vitro tubulin polymerization assay*

A tubulin polymerization assay was performed by monitoring the ﬂuorescence enhancement due to the integration of a ﬂuorescence reporter into microtubules as polymerization happens. In this study, we have employed a commercial kit (cytoskeleton, cat. #BK011P) for the tubulin polymerization (21). The final buffer used for tubulin polymerization contained 80.0 mM piperazine-N,N’ -bis(2- ethanesulfonic acid) sequisodium salt (pH 6.9), 2.0 mM MgCl2, 0.5 mM EGTA, 1.0 mM GTP, and 10.2% glycerol. First, 5 µl of the tested compound at different concentrations, Paclitaxel (a polymerization promoter) at 3 µM concentration and CA4 (a polymerization suppressor) at 5 µM concentration, were poured into wells of a 96-well plate, and the mixture was warmed to 37 °C for 1 min; then, the reaction was initiated by the addition of 55 µl of the tubulin solution. Polymerization was measured by excitation at 360 nm and emission at 420 nm for 1 hr at 1 min intervals in a plate reader (Synergy H4, USA). 

*Cell cycle analysis using flow cytometry*

Flow cytometric analysis (FACS) was performed to calculate the distribution of the cell population with propidium iodide (PI) through the cell cycle phases. Tumor cells (2.5 x 105 cells) were seeded in 6-well cell culture plates for 24 hr, then treated with different concentrations of the compounds **5g, 5c,** and **4d**, and vehicle alone (0.05% DMSO). Treated and untreated cells were incubated for 48 hr, washed with PBS and ﬁxed with 70% ethanol, then washed twice with PBS, and incubated for 0.5 hr at 37 °C in a PBS solution containing 0.1 mg/mL RNase A and propidium iodide. The data regarding the number of cells in different phases of the cell cycle were analyzed using the Flowjo software package.

***Molecular docking***

The molecular docking process was performed using AutoDock4.2(http://autodock.scripps.edu/). The x-ray crystal structure of tubulin (PDB ID:4O2B) with resolution 2.5 A° was downloaded from the Protein Data Bank (PDB). Water molecules, co-crystallized ligand, and all ions were removed and polar hydrogen atoms were added to the receptor, then the protein was saved in the pdbqt format using the graphical user interface Autodock tools (ADT, 1.5.6). The 2D structure of the target compounds was prepared using Chem Draw Ultra 8.0 software (http://www.cambridgesoft.com/) and converted to 3D format by HyperChem7 (Hyper cube Inc, USA) using AM1 semi-empirical method, and the pdbqt format of the target compounds was prepared using ADT. The target compounds were docked into the active site of tubulin. A grid box size of 50 ×50 ×50 points with 0.375 A° spacing between the grid points was used. Grid box center was located at center of co-crystallized ligand (x =15.761, y =13.765, z =‒17.608). AutoGrid 4.2 was used to generate the grid map files for the docking calculations. Then AutoDock4.2 was run. Lamarckian Genetic Algorithm (GA) parameters were set to 100 GA runs with a population size of 150; a maximum number of 2.5 × 105 energy evaluation and 2.7 × 104 generation were used. The other parameters were set as default. The output DLG files were converted to pdb format and Molecular Operating Environment (MOE) (www.chemcomp.com) was employed to view the docking results.

**Table 1:** *In vitro *antiproliferative activities (IC50 (µM) a) of synthesized compounds and CA-4 against human cancer cell lines

**Compound**	**R**_1_	**R**_2_	**R**_3_	**R**_4_	**X**	**MCF-7**	**MCF7-MX**	**A2780**	**A2780-RCIS**
**4a**	H	CH_3_	H	H	C	>100	75.5±5.21	>100	58.72±4.33
**4b**	H	OCH_3_	H	H	C	ND	ND	ND	ND
**4c**	OCH_3_	OCH_3_	H	H	C	>100	>100	31.5±2.35	46.7±3.89
**4d**	OCH_3_	OCH_3_	OCH_3_	H	C	ND	47.6±3.94	24.3±2.78	52.8±3.98
**4e**	OH	OCH_3_	H	H	C	26.87±3.67	15.31±2.91	23.47±1.84	14.73±2.34
**4f**	OCH_3_	OCH_3_	OCH_3_	H	N	ND	ND	ND	ND
**5a**	H	CH_3_	H	CH_3_	C	30.74±3.84	40.9±4.24	24.3±2.63	32.6±3.84
**5b**	OCH_3_	OCH_3_	H	CH_3_	C	33.43±3.71	>100	26.91±2.72	31.4±2.56
**5c**	OCH_3_	OCH_3_	OCH_3_	CH_3_	C	32.5±3.21	9.05±1.83	12.2±1.45	18.3±2.23
**5d**	H	CH_3_	H	benzyl	C	100±4.91	ND	31.5±3.25	28.32±2.45
**5e**	H	OCH_3_	H	benzyl	C	98.11±4.56	70.9±4.76	20.7±2.11	25.68±2.17
**5f**	OCH_3_	OCH_3_	H	benzyl	C	30.3±2.44	71.10±5.36	18.70±1.84	24.4±2.16
**5g**	OCH_3_	OCH_3_	OCH_3_	benzyl	C	11.17±1.57	20.81±2.15	4.47±1.14	19.88±2.19
**CA4**						1.23±0.42	2.97±0.41	2.84±0.27	2.95±0.61

^a^ compound concentration required to inhibit tumor cell proliferation by 50%. Data are presented as the mean±SD from the dose−response curves of three independent experiments

**Figure 2:**
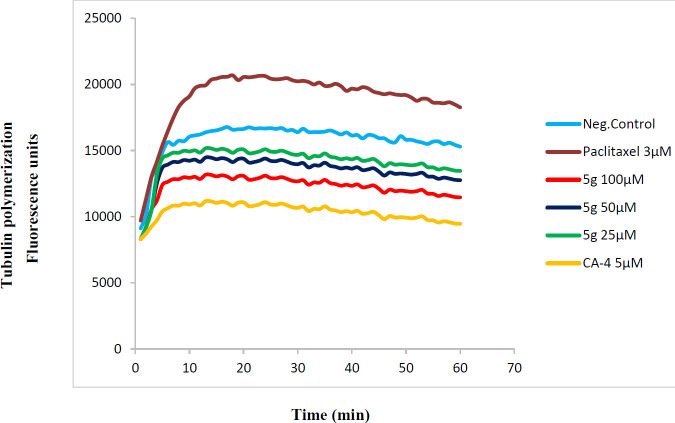
Effect of compounds 5g on *in vitro* tubulin polymerization

**Figure 3:**
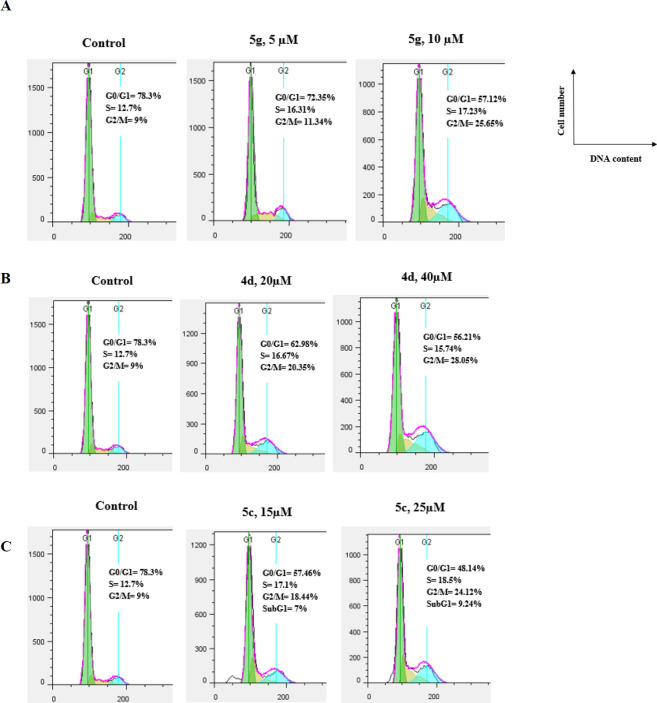
Flow cytometry analysis of compound 5g (A), 4d (B), and 5c (C) in A2780 cells

**Figure 4:**
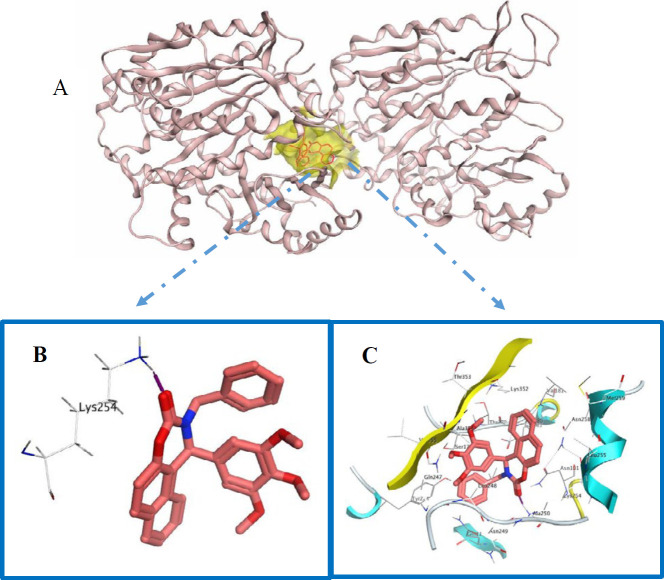
(A) compound **5g** in colchicine binding site of tubulin. (B and C) Hydrogen and Hydrophobic interactions of **5g** with colchicine binding site of tubulin

## Results

***Synthesis***

As illustrated in scheme 1, β-naphthol, substituted benzaldehydes and urea were combined in the presence of a catalytic amount of acetic acid under microwave irradiation to obtain the target compounds **4a-4e**, then benzyl or methyl derivatives** 5a-5g **were prepared using benzyl bromide or methyl iodide in the presence of sodium hydride. The compounds were characterized by nuclear magnetic resonance and infrared spectrometry.

***Biological evaluation***

*In vitro anticancer activity*

The cytotoxic activity of the synthesized compounds was evaluated against MCF-7, A2780, MCF-7/MX, A2780/RCIS, employing MTT assay using combretastatin A-4 as the positive control. As illustrated in Table 1, in general, the benzylic derivative **5g** showed the most cytotoxic effect in cancer cells in comparison with other compounds. The simplest compound, **4a**, did not show significant cytotoxic effect in cancer cells. Inserting three methoxy groups in ring A of **4a**, increased the cytotoxicity on MCF-7/MX, A2780, and A2780RCIS cancer cells (compare the cytotoxic activity of compounds **4a** and **4d**). Compound **4e** possessing 3-hydroxy-4-methoxyphenyl moiety showed significant cytotoxic effects in all cancer cell lines. In general, N-methyl and N-benzyl derivatives of **4a-4f **demonstrated more cytotoxicity against all cancer cells in comparison with their parents **4a-4f**, this may be due to better ability of **5a-5g** to penetrate the cell membrane because of their high lipophilicity or better interaction of these compounds with tubulin. Compounds **5a**, **5b**, and **5c**, N- methyl derivatives of **4a**, **4c**, **4d**, and compounds **5d** and **5g**, N-benzyl derivatives of **4a** and **4d** showed also more cytotoxicity in comparison with their parents. Replacing the methyl group of **5d** with the methoxy group, increased the cytotoxicity, significantly (compare the cytotoxic activity of compounds **5d** and **5e**). Also, in compounds **5d-5g** with increasing the number of methoxy groups on the A ring, the cytotoxic activity of the compounds increased (**5g**>**5f**>**5e**>**5d**).

*Tubulin polymerization assay*

To examine whether the antiproliferative activity of compounds was due to their binding to tubulin, compound **5g**, which demonstrated the most anti-proliferative activity, was selected to test the inhibitory effect on microtubule assembly *in vitro*, CA-4 as the polymerization suppressor and paclitaxel as the polymerization promoter were used (22). For characterization of tubulin polymerization, the fluorescence intensity was examined every minute and the results were shown in Figure 2. Compound **5g** at 25-100 µM concentrations inhibited tubulin polymerization in a dose-dependent manner and in a manner similar to that of CA-4 (5 µM).

*Cell cycle analysis using ﬂow cytometry*

The anti-mitotic drugs arrest cell cycle at the G2/M phase in cancer cells due to damage of the microtubular cytoskeleton(23). We examined the effect of the compounds **5g**, **5c**, and **4d** on the cell cycle using the propidium iodide staining method (24). A2780 cells were treated with increasing concentrations of compounds **5g**, **5c**, and **4d** for 48 hr. They were stained with propidium iodide and then analyzed by flow cytometry. A2780 cells treatment with compound **5g** at 5 and 10 µM, displayed enhancement in the G2/M population from 9% (control) to 11.34% and 25.65%, respectively in a dose-dependent manner (Figure 3). 

**4d** caused G2/M arrest in a concentration-dependent manner. Only 9% of the A2780S cells were arrested in G2/M phase in the control group after 48 hr treatment, the percentage of G2/M phase increased to 20.35% and 28.08% when cells were exposed to 20 µM and 40 µM of **4d**. Similarly, the cell accumulation percentage at the G2/M phase increased from 9% (control) to 18.44% and 24.12 % with a concentration rising from 15 to 25 µM of **5c**. The results suggest that **5g**, **4d**, and 5c induced cell cycle arrest in the G2/M phase in A2780S cells. 

*Molecular modeling studies*

In order to investigate the binding mode of the target compounds in the catalytic site of the tubulin molecular docking studies were carried out on the synthesized oxazinonaphthalene-3-one analogs and co-crystallized ligand against the colchicine binding site of the tubulin crystal structure (PDB ID: 4O2B) using Auto Dock 4.2 (25). 

DThe docking study showed that the top-ranked conformation of all the selected compounds was well accommodated inside the colchicine binding site of the tubulin. Compound **5g** has shown one hydrogen bond interaction between the carbonyl group with residues Lys 254β (Figure 4 B). In addition, compound **5g** is surrounded by hydrophobic residues like Met 259β, Ile 378β, Ala 180, Cys 241β, Leu 242β, Lys 352β, Thr 353β, and Leu 248β. These hydrogen bonds and hydrophobic interactions can describe tubulin inhibitory effects of this compound. 

## Discussion

In the current study, a new series of oxazinonaphthalene-3-one analogs were synthesized and evaluated for their *in vitro *antiproliferative activities by MTT assay against four human cancer cell lines including MCF-7, MCF-7/MX, A-2780, and A-2780/RCIS. In general, **5a-5g**, N-methyl and N-benzyl derivative of **4a-4f **demonstrated more cytotoxicity against all cancer cells in comparison with their parent **4a-4f**, this may be due to the better ability of **5a-5g** to penetrate the cell membrane because of their high lipophilicity or better interaction of these compounds with tubulin. Among them, **4d**, **5c**, and **5g** possessing trimethoxy groups in the A ring showed significant cytotoxic activity with IC_50_ values ranging from 4.4-52.8 μM stronger than the other compounds. The cell cycle analysis indicated that **4d**, **5c**, and **5g** disrupted the microtubule network and arrested cells in the G2/M phase of the cell cycle in the A2780 cancer cell line.

## Conclusion

Among the synthesized compounds, **5g** possessing trimethoxy groups and N-benzyl group were found to be the most potent cytotoxic agents against four human cancer cell lines with an IC_50 _value of 4.47–20.81 µM. Also, compound **5g** inhibited tubulin polymerization in a dose-dependent manner and arrested cells in the G2/M phase of the cell cycle in the A2780 cancer cell line. Finally, molecular docking studies of **5g** into the colchicine-binding site of tubulin represented the possible interaction of this compound in the active site of tubulin. These results suggested that oxazinonaphthalene-3-one derivatives could be promising lead compounds for further development of anticancer agents through inhibition of tubulin.

## References

[1] Chinigo GM, Paige M, Grindrod S, Hamel E, Dakshanamurthy S, Chruszcz M (2008). Asymmetric synthesis of 2, 3-dihydro-2-arylquinazolin-4-ones: methodology and application to a potent fluorescent tubulin inhibitor with anticancer activity. J Med Chem.

[2] Hamel E (1996). Antimitotic natural products and their interactions with tubulin. Med Res Rev.

[3] Honore S, Pasquier E, Braguer D (2005). Understanding microtubule dynamics for improved cancer therapy. Cell Mol Life Sci.

[4] Mirzaei S, Hadizadeh F, Eisvand F, Mosaffa F, Ghasemi A, Ghodsi R (2020). Design, synthesis and biological evaluation of novel 5, 6, 7-trimethoxy-N-aryl-2-styrylquinolin-4-amines as potential anticancer agents and tubulin polymerization inhibitors. Bioorg Chem.

[5] Behbahani FS, Tabeshpour J, Mirzaei S, Golmakaniyoon S, Tayarani-Najaran Z, Ghasemi A (2019). Synthesis and biological evaluation of novel benzo[c]acridine-diones as potential anticancer agents and tubulin polymerization inhibitors. Arch Pharm.

[6] Mirzaei S, Hadizadeh F, Eisvand F, Mosaffa F, Ghodsi R (2020). Synthesis, structure-activity relationship and molecular docking studies of novel quinoline-chalcone hybrids as potential anticancer agents and tubulin inhibitors. J Mol Struct.

[7] Karimikia E, Behravan J, Zarghi A, Ghandadi M, Omid Malayeri S, Ghodsi R (2019). Colchicine-like β-acetamidoketones as inhibitors of microtubule polymerization: Design synthesis and biological evaluation of in vitro anticancer activity. Iran J Basic Med Sci.

[8] Romagnoli R, Baraldi PG, Carrion MD, Cruz-Lopez O, Cara CL, Basso G (2009). 2-Arylamino-4-amino-5-aroylthiazoles “One-pot” synthesis and biological evaluation of a new class of inhibitors of tubulin polymerization. J Med Chem.

[9] Cushman M, Nagarathnam D, Gopal D, Chakraborti AK, Lin CM, Hamel E (1991). Synthesis and evaluation of stilbene and dihydrostilbene derivatives as potential anticancer agents that inhibit tubulin polymerization. J Med Chem.

[10] Ohsumi K, Hatanaka T, Fujita K, Nakagawa R, Fukuda Y, Nihei Y (1998). Syntheses and antitumor activity of cis-restricted combretastatins: 5-membered heterocyclic analogues. Bioorg Med Chem Lett.

[11] Pettit GR, Rhodes MR, Herald DL, Hamel E, Schmidt JM, Pettit RK (2005). Antineoplastic agents Synthesis and evaluation of structural modifications of (Z)- and (E)-combretastatin A-41. J Med Chem.

[12] Hsieh HP, Liou JP, Mahindroo N (2005). Pharmaceutical design of antimitotic agents based on combretastatins. Curr Pharm Des.

[13] Nam NH (2003). Combretastatin A-4 analogues as antimitotic antitumor agents. Curr Med Chem.

[14] Tron GC, Pirali T, Sorba G, Pagliai F, Busacca S, Genazzani AA (2006). Medicinal chemistry of combretastatin A4: present and future directions. J Med Chem.

[15] Pinney KG, Jelinek C, Edvardsen K, Chaplin DJ, Pettit GR (2005). The discovery and development of combretastatins in anticancer agents from natural products; Gordon M. Cragg, David G. I. Kingston, David J. Newman (Eds). Taylor & francis group boca raton florida.

[16] Lopus M, Oroudjev E, Wilson L, Wilhelm S, Widdison W, Chari R (2010). Maytansine and cellular metabolites of antibody-maytansinoid conjugates strongly suppress microtubule dynamics by binding to microtubules. Mol Cancer Ther.

[17] Ghodsi R, Azizi E, Grazia Ferlin M, Pezzi V, Zarghi A (2016). Design, synthesis and biological evaluation of 4-(imidazolylmethyl)-2-aryl-quinoline derivatives as aromatase inhibitors and anti-breast cancer agents. Lett Drug Des Discov.

[18] Jafari F, Baghayi H, Lavaee P, Hadizadeh F, Soltani F, Moallemzadeh H (2019). Design, synthesis and biological evaluation of novel benzo-and tetrahydrobenzo-[h] quinoline derivatives as potential DNA-intercalating antitumor agents. Eur J Med Chem.

[19] Malayeri SO, Abnous K, Arab A, Akaberi M, Mehri S, Zarghi A (2017). Design, synthesis and biological evaluation of 7-(aryl)-2, 3-dihydro-[1, 4] dioxino [2, 3-g] quinoline derivatives as potential Hsp90 inhibitors and anticancer agents. Bioorg Med Chem.

[20] Nakagawa-Goto K, Taniguchi Y, Watanabe Y, Oda A, Ohkoshi E, Hamel E (2016). Triethylated chromones with substituted naphthalenes as tubulin inhibitors. Bioorg Med Chem.

[21] Pang Y, Yan J, An B, Huang L, Li X (2017). The synthesis and evaluation of new butadiene derivatives as tubulin polymerization inhibitors. Bioorg Med Chem.

[22] Xu S, An B, Li Y, Luo X, Li X, Jia X (2018). Synthesis and evaluation of new 2-chloro-4-aminopyrimidine and 2, 6-dimethyl-4-aminopyrimidine derivatives as tubulin polymerization inhibitors. Bioorg Med Chem Lett.

[23] Guggilapu SD, Guntuku L, Reddy TS, Nagarsenkar A, Sigalapalli DK, Naidu V (2017). Synthesis of thiazole linked indolyl-3-glyoxylamide derivatives as tubulin polymerization inhibitors. Eur J Med Chem.

[24] Swami R, Singh I, Jeengar MK, Naidu V, Khan W, Sistla R (2015). Adenosine conjugated lipidic nanoparticles for enhanced tumor targeting. Int J Pharm.

[25] Ravelli RB, Gigant B, Curmi PA, Jourdain I, Lachkar S, Sobel A (2004). Insight into tubulin regulation from a complex with colchicine and a stathmin-like domain. Nature.

